# Predicting Apnoeic Events in Preterm Infants

**DOI:** 10.3389/fped.2020.00570

**Published:** 2020-09-16

**Authors:** Kathleen Lim, Haimin Jiang, Andrew P. Marshall, Brian Salmon, Timothy J. Gale, Peter A. Dargaville

**Affiliations:** ^1^Menzies Institute for Medical Research, College of Health and Medicine, University of Tasmania, Hobart, TAS, Australia; ^2^Neonatal and Pediatric Intensive Care Unit, Royal Hobart Hospital, Hobart, TAS, Australia; ^3^School of Engineering, College of Science, Engineering and Technology, University of Tasmania, Hobart, TAS, Australia

**Keywords:** apnoea of prematurity, preterm infants, prediction, machine learning, neonatal intensive care

## Abstract

Apnoea, a pause in respiration, is almost ubiquitous in preterm infants born before completing 30 weeks gestation. Apnoea often begets hypoxemia and/or bradycardia, and has the potential to result in adverse neurodevelopmental consequences. Our current inability to predict apnoeic events in preterm infants requires apnoea to first be detected by monitoring device/s in order to trigger an intervention by bedside (medical or nursing) staff. Such a reactive management approach is laborious, and makes the consequences of apnoeic events inevitable. Recent technological advances and improved signal processing have allowed the possibility of developing prediction models for apnoeic events in preterm infants. However, the development of such models has numerous challenges and is only starting to show potential. This paper identifies requisite components and current gaps in developing prediction models for apnoeic events, and reviews previous studies on predicting apnoeic events in preterm infants.

## Introduction

Respiratory immaturity is a regular accompaniment of premature birth, and is a major contributor to the protracted requirement of respiratory support during the first weeks of life in a neonatal intensive care unit (NICU) (1–4). Apnoea, defined as a respiratory pause of ≥ 20 s, or a respiratory pause of ≥10 s associated with hypoxia (oxygen saturation <80%) and/or bradycardia (heart rate <100 beats per minute), is almost ubiquitous in preterm infants (1, 2, 5). Apnoeic events are most prominent in weeks 2 to 4 of life, when infants are usually managed on non-invasive respiratory support, and is observed in essentially all infants born at <28 weeks gestation, and in up to 85% of infants born between 28 to 34 weeks gestation (1–4). There are three main types of apnoea; central apnoea where respiratory drive is lost; obstructive apnoea where ventilation is impeded by mechanical obstruction of the upper airways; and mixed apnoea (a combination of central and obstructive apnoea). Whatever the form, apnoea often leads to hypoxemia and/or bradycardia, which cumulatively have the potential to result in adverse long term consequences, including retinopathy of prematurity, and neurodevelopmental impairment (6).

Current management of preterm infants with prominent apnoea includes caffeine administration to regularize central respiratory drive (7), and application of continuous positive airway pressure to stimulate and splint the upper airway (7, 8). Despite these therapies, apnoeic events may continue to occur throughout an infant's NICU admission, requiring bedside staff to provide urgent interventions, often only after having observed apnoea-associated hypoxia and bradycardia, to re-establish respiration (4, 9). This labor-intensive reactive management system is in part a consequence to our inability to reliably predict apnoeic events in real time in preterm infants.

The ability to predict and be forewarned of impending apnoeic events in preterm infants has the potential to provide nurses with the opportunity to take a proactive approach in apnoea management. This means that an early alarm system could allow nurses to provide a stimulus prior to the onset of apnoea in an attempt to maintain respiratory cadence, or otherwise to limit the duration and side effects of apnoea. A reliable apnoea prediction system could be further developed by linkage to an automated stimulus to mitigate the consequences of apnoea.

## Prediction of Apnoea—The Challenge

Predicting the occurrence of physiological events requires identification of a change in state within an adequate time window prior to the event. Anticipatory data processing in medicine involves managing a continuous input of physiological signals which are often complex, and have a high inter-patient variability (10). The prediction of a medical event is further complicated by the need to differentiate normal intra- and inter-patient variations from changes which herald an event. The baseline physiological state of a preterm infant changes within a short timeframe with maturation, progression of health conditions, and response to treatments, creating a time dependence when attempting to predict impending apnoeic events. Given that the prediction of apnoeic events in preterm infants needs to take into account multiple factors, some of which have a continuously shifting baseline, this task is beyond the scope of individual clinicians or bedside staff.

With a large, complex, and variable input dataset, and without any known algorithms to predict apnoeic events in preterm infants, machine learning systems have been investigated for their potential to develop a predictive model for apnoeic events. Different machine learning systems have been previously tested to predict apnoeic events in both adults and preterm infants with varying success (11–16).

This paper identifies some requisite components and current gaps in developing prediction models for apnoeic events, and reviews previous methods and outcomes of apnoea prediction in preterm infants.

## Requisite Components for a Real Time Apnoea Prediction System

### Machine Learning Techniques

**Figure 1:**
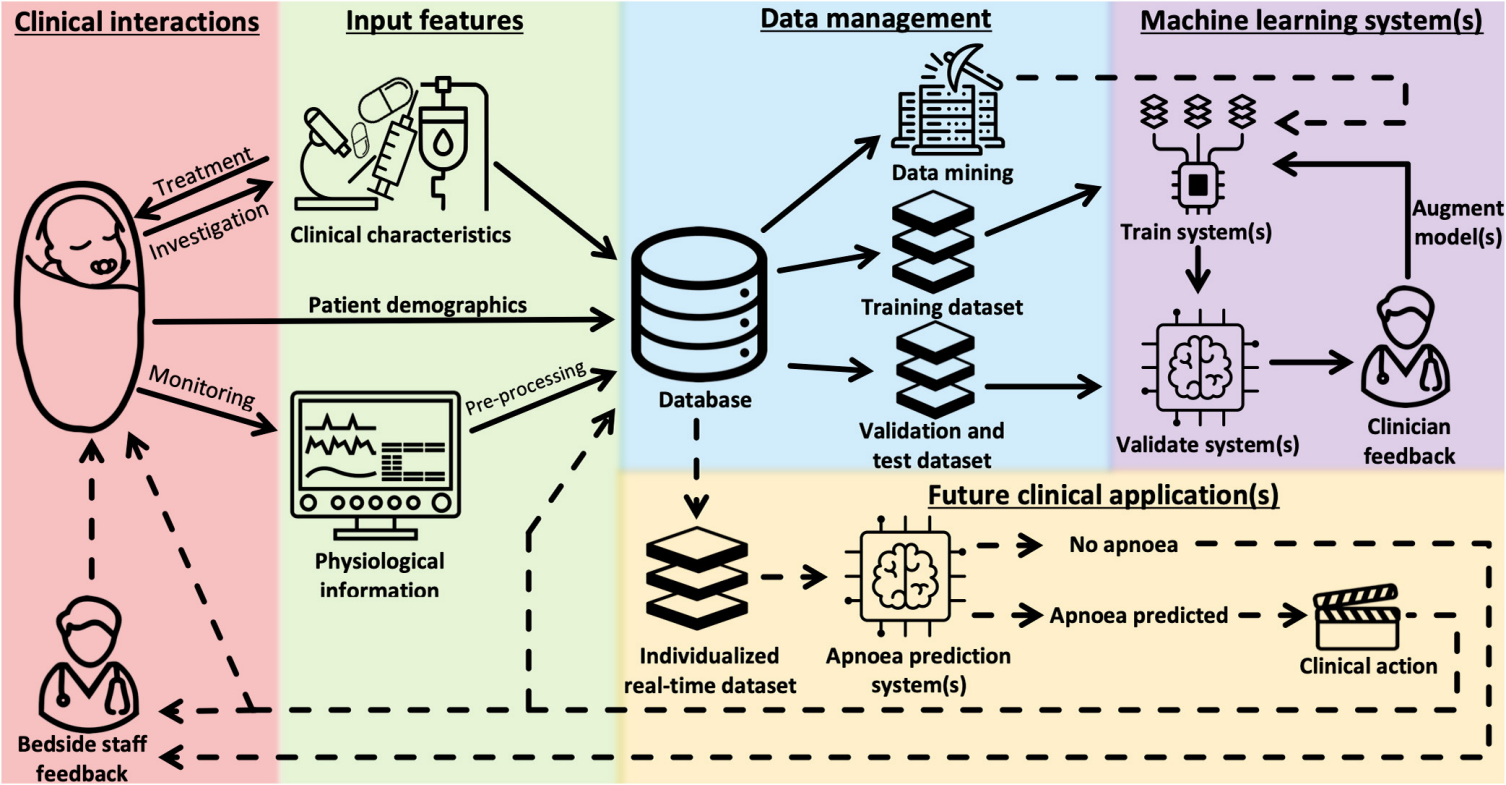
Development of a machine learning-based system for prediction of apnoeic events in preterm infants. Solid lines, Steps already undertaken to develop a predictive system; Dashed lines, Future steps for development and application of a machine learning system to predict apnoeic events. *Patient demographics*: may include birth gestation, postnatal age, birth and current weight, Apgar scores, and medical diagnoses. *Clinical characteristics*: may include mode, duration and level of respiratory support, dosage and timing of previous and current treatment (e.g., surfactant therapy, post-natal corticosteroid, caffeine), and investigation findings (e.g., hemoglobin, radiographic findings). *Physiological information*: may include heart rate and R-R interval, respiratory rate and inter-breath interval, movement patterns, and characteristics of previous apnoeic events (e.g., type and duration of event, latency between events, apnoea-associated destabilization). *Clinical action*: may include an early warning display, a servo-controlled stimulus to maintain respiratory cadence, or decision support regarding further preventative management.

Machine learning involves the science of developing algorithms to derive models for a specific task, by recognizing complex patterns using supplied inputs to infer meaning (17, 18). Machine learning methods are divided into two categories, namely, supervised and unsupervised. A supervised approach requires user-defined outputs (target classes) to derive correlation among inputs and defined outputs. Training, validation, and test datasets are used to guide the process of producing reduced generalization errors in output by the machine learning system (Figure 1) (17).

Artificial neural networks (ANNs) are a matured technology used in various fields to manage more complex tasks (17). An ANN consists of densely interconnected processing elements (artificial neurons) in a layered architecture, which adjusts inputs using defined weighting synaptic matrices with biases to generate desired outputs with minimal errors (17, 19). A validation dataset is used to further refine the ANN, and synaptic weight changes as new data are being processed, in order to produce reliable classifications with optimal generalization (20). With advanced computing power, training machine learning systems with large datasets is feasible within a reasonable timeframe. This has led to a surge in application of ANNs across different fields, such as classifying images (e.g., Google Images) and speech recognition (e.g., Apple's Siri).

To further enhance the predictive accuracy of machine learning systems, different machine learning techniques can be used in combination (i.e., ensemble methodology) to compensate for the types of errors associated with each machine learning technique when used individually (17). An ensemble method fine-tunes a machine learning system by aggregating predictions of different machine learning techniques to decide on the final output, allowing a higher level of accuracy in the predicted outcome (17).

The application of machine learning to apnoea prediction is in its infancy, and in the next sections we examine the target classes and input features relevant to an ANN used for this purpose.

### Target Classes in Apnoea Prediction

To predict apnoeic events in preterm infants using a machine learning system, respiratory recordings would be divided into four target classes based on proximity to an apnoeic event: pre-apnoea, apnoea, post-apnoea, and non-apnoea. To obtain a clinically meaningful prediction, the definition of each target class needs to be carefully considered, and should take account of current definitions of apnoea (5, 21) but also be clinically relevant.

### Input Features to Predict Impending Apnoeic Events in Preterm Infants

Standard clinical and physiological monitoring parameters can be used as input features for analysis by the machine learning system to predict impending apnoeic events in preterm infants (Figure 1). These input features should be selected with the aim of improving accuracy of the machine learning system when used in real time.

Several routinely monitored physiological changes have previously been linked to apnoeic events in preterm infants, and have shown potential predictive value in identifying a pre-apnoeic state (14, 22–24). These physiological parameters include respiratory rate and pattern, as well as oxygen saturation changes within a 2 h window preceding an apnoeic event (24). A lower-frequency oscillation respiratory pattern in infants was also found to be associated with longer apnoeic events (22). The preterm infant's respiratory control system matures with age and is associated with a shifting physiological baseline (25). The distribution density of inter-breath intervals (IBIs) in preterm infants was described to follow a power law, and breathing patterns have been found to increase in complexity as the infant's respiratory control center matures (25, 26). Therefore, prediction models for apnoeic events in preterm infants need to account for the expected respiratory physiological changes related to maturation.

In addition to respiratory parameters, progressive changes in heart rate and heart rate variability (R-R interval) have also been identified to precede apnoeic events (14, 22–24). Preterm infants with persistent apnoea reportedly had a lower heart rate, and an increased R-R interval when compared to healthy infants (23, 24, 27). Furthermore, changes in movement patterns were also identified to precede apnoeic events in preterm infants (28, 29). Body movement could be part of a transient arousal and associated with hyperventilation and hypocapnia, ultimately reducing chemoreceptor stimulation, suppressing central neural respiratory drive and resulting in central apnoea (14, 29).

Heart rate, R-R interval variability, respiratory rate, IBIs variability, and movement pattern of preterm infants have been proposed as input features for identifying a pre-apnoeic state (14, 15). Using physiological input features to predict onset of apnoeic events in preterm infants allows for adaptation on a per patient basis, overcoming the imprecisions introduced by inter-patient variation, and potentially allowing an individualized machine learning system to be implemented for each infant (14).

Raw physiological data often contain noise and artifacts (including motion artifact) which needs to be pre-processed prior to being used by a machine learning system (17). By pre-processing the input data, effectiveness in developing a predictive model is increased by removing signal artifact and emphasizing properties of input features with potentially discriminative values (14). Furthermore, deriving features reduces the search space, and standardization of input features with reference to the patient's baseline would significantly reduce the complexity of the model used by the predictive system (10, 14, 15).

Methods of pre-processing are defined by the type of application and input data used. Generally, this involves filtering and managing signal artifacts (i.e., missing data), scaling data (through standardization or normalization), transforming (manifold adjustment), and/or aggregation (collating multiple features into a single input feature) (14, 15, 17).

## Previous Studies of Apnoeic Event Prediction

### Predicting Apnoeic Events in Adults With Obstructive Sleep Apnoea (OSA)

The prediction of apnoeic events has been previously applied and studied in adults with OSA, utilizing machine learning systems. Using various forms of ANN, apnoeic events in adults have been predicted with a sensitivity and specificity of up to 80.6 and 72.8% respectively (11–13). Maali et al. compared different ANNs for their effectiveness in predicting apnoeic events in adults with OSA, and recommended the use of an ensemble of ANNs with a dynamic model selection (13). This approach allows the selected ANN used to predict apnoeic events to evolve in real time, corresponding to the best testing sample resulted from amongst all the ANNs within the ensemble, in order to achieve a better predictive model (13). The study by Maali et al. provided evidence that the ideal ANN for predicting apnoeic events needs to be individualized for each patient, and will be influenced by the selected input features, as well as the different prediction and segmentation time windows (13).

While adults with OSA have a more stable respiratory physiology than preterm infants, the concept and components of apnoea prediction in adults can potentially be extrapolated and applied to preterm infants.

### Predicting Apnoeic Events in Preterm Infants

Studies regarding the application of machine learning techniques in preterm infants have been undertaken *in silico*, with the aim of predicting apnoeic events to develop an early warning system for bedside staff (14–16). The first published attempt at predicting individual apnoeic events in preterm infants used a mathematical technique—equal prior quadratic classifier—to separate the measurements of different target classes (15). Several other machine learning techniques have since been evaluated to predict apnoeic events in preterm infants, achieving an accuracy (proportion of true positive and true negative predictions) of (up to) 88% (Table 1) (14–16).

**Table 1:** Summary of published machine learning systems for prediction of apnoeic events in preterm infants.

**Author**	**Sample size,** **gestational age (weeks),** **Birthweight (g)**	**Study duration (mins),** **No. of apnoeic event**	**Respiratory detection**	**Input features**	**Pre-processing technique**	**Machine learning technique**	**Pre-apnoea window (mins)**	**Evaluation**
Williamson et al. (15)	6, 28.6, 1,240	2015,34	Abdominal respiratory inductance plethysmography	Inter-breath intervalR-R interval	Linear interpolation Log transformation Conversion to standard units for each patient	Equal prior quadratic classifier	7.5	^*^AUC = 0.73, *p* = 0.13
Williamson et al. (14)	6, 28.6, 1,240	2015,34	Abdominal respiratory inductance plethysmography	Inter-breath intervalR-R intervalMovement features	Linear interpolation Log transformation Conversion to standard units for each patient	Bayesian adaptation of Gaussian mixture models	7.5	^*^AUC = 0.80, *p* = 0.00
Shirwaikar et al. (16)	299, ND, ND	ND,ND	Not stated	23 features recorded including demographic, maternal co-variates and physiological input	Transformation Normalization	Decision tree (C5.0)	ND	Accuracy = 0.75, Sensitivity = 0.20, Specificity = 0.88
						Support vector machine using radial kernel		Accuracy = 0.75, Sensitivity = 0.28, Specificity = 0.72
						Ensemble approach: ∙ Bagged decision tree ∙ Auto tuned boosted C5.0 ∙ Random forest		Accuracy = 0.88, Kappa = 0.72

*Gestational age and birthweight are presented as mean only*.

* *Area under the receiver operating characteristic curve; AUC, Area under curve*.

*ND, Not described*.

The methods used to pre-process input data and definitions of target classes for predicting apnoeic events in preterm infants have been most clearly described by Williamson et al. (14, 15). In the pre-processing stage, these authors initially performed a linear interpolation, and subsequently cubic interpolation, of physiological features, followed by log transformation to make R-R intervals and IBIs normally distributed (14, 15). For target class labeling, apnoeic events were defined as per the established consensus—a respiratory pause of ≥20 s, or a respiratory pause of ≥10 s if associated with bradycardia and/or hypoxia (5, 14, 15). Pre-apnoea was then defined by Williamson et al. as the 7.5 min duration preceding an apnoeic event, and post-apnoea defined to be the 7.5 min period following an event. All other sections of the recordings were labeled as non-apnoea (14, 15). While these definitions of target classes by Williamson et al. provided a starting point, 7.5 min is a relatively long time window for pre-apnoea, and would need to be shortened in order to provide a more effective early warning system that could reasonably be integrated into current clinical practice.

Thus far, studies predicting apnoea events in preterm infants have only been conducted *in silico*, applying machine learning to pre-recorded physiological data from preterm infants. Further investigations of different machine learning techniques applied to larger datasets (i.e., larger sample size with longer duration of recording per infant) are still required to further our understanding and selection of the most appropriate input features, the predictive time window and the machine learning technique, or ensembles thereof, for predicting apnoeic events in preterm infants (14, 15).

## Current Gaps

### Pre-processing Input Features in Real Time

The quality and type of input features provided to the machine learning system is paramount to effective and accurate prediction of apnoeic events. The quality of physiological data obtained from preterm infants is commonly compromised, mostly by motion artifact, but also by therapeutic manoeuvers, interference from other electrical equipment and ambient light (30–32). While such disturbances can be identified and potentially excluded when pre-processing the dataset used to train the machine learning system, a method of excluding artifacts when applying the machine learning system in real time is required to maintain the effectiveness, and accuracy, of predicting apnoeic events.

### Types of Input Features Used

IBIs, R-R intervals, and changes in movement patterns are the main input features previously used to predict apnoeic events, and are physiological measures routinely monitored in preterm infants admitted to the NICU (14, 15). Other potential input features, such as electroencephalography and electromyography, have yet to be explored for their discriminative values as input features in prediction of apnoeic events in preterm infants (33, 34). Additionally, no studies have reported the application of clinical information (i.e., caffeine dosage, gestational age, etc.) as input features for machine learning systems to predict apnoeic events, despite studies demonstrating an inverse relationship between age and frequency of apnoea (1, 2, 4).

In practice, the selection of input features used to predict apnoeic events in preterm infants is an ongoing process requiring repeated iterations, whereby the machine learning system is repeatedly refined with different inputs, to improve its performance based on *in silico* trials and clinicians' observations and feedback (Figure 1).

### Choice of Machine Learning System

Previous studies have examined a subset of machine learning techniques (Table 1); however, there are many other established approaches yet to be examined either individually or in an ensemble method for preterm infants (14–16).

Furthermore, previously examined machine learning techniques approached the prediction of apnoeic events as a classification problem, where discrete categories of respiratory phases were identified. Beyond the identification of impending apnoeic events, prediction algorithms could be further advanced by approaching the task as a regression problem, which allows additional features of an apnoeic event to be forecast, such as the type of apnoea (i.e., obstructive, central or mixed), predicted duration and physiological impact of an impending apnoeic event. A qualitative output would be able to better direct the appropriate preventative or therapeutic stimuli required for each predicted apnoeic event.

### Need for Large Dataset

Previous studies have examined machine learning techniques based on a small dataset of six preterm infants with 2015 min of data and 34 apnoeic events (14, 15). While Shirwaikar et al. used a dataset obtained from 299 infants, but the duration of data collected from each infant, and the number of apnoeic events examined were unclear (16). Using data with recordings from a larger number of preterm infants, as well as a longer duration of recording per infant would allow a more generalizable machine learning system to be developed. Such a system would subsequently provide an efficient platform upon which to build the component of adaptive learning when applied in real time to individual preterm infants.

## Conclusion

The ability to accurately predict upcoming apnoeic events would provide an opportunity to improve current apnoea management in preterm infants, and in turn decrease the long term adverse consequences associated with apnoea (Figure 1). An early warning system for apnoeic events would allow preventative or therapeutic stimuli to be delivered sooner, with the aim of maintaining respiratory cadence and/or curtailing the duration and immediate physiological effects of apnoea.

As with any form of prediction model used in healthcare, the impact of mispredicting apnoeic events in preterm infants needs to be considered, and appropriate safety mechanisms will be required. When prediction models for apnoeic events in preterm infants are used independently only as a warning system, a high false positive prediction rate would increase the risk of alarm fatigue amongst bedside staff, while a high false negative rate would nullify the value of the predictive tool. If prediction models were to be coupled with automated delivery of preventative or therapeutic stimuli for apnoea, false positive prediction would result in delivery of unnecessary stimuli.

The development of anticipatory medicine, and use of machine learning systems in healthcare, particularly in preterm infants, is still in its early stages, and further studies of various prediction models based on larger datasets are required. While there are challenges with predicting impending apnoeic events in preterm infants, these challenges can be surmounted by incorporating solutions found through application of machine learning in other fields.

## Author Contributions

KL wrote the first draft of the manuscript and approved the final draft. HJ, AM, BS, TG, and PD participated in contributing to, critically reviewing and editing the manuscript, and approved the final draft.

## Conflict of Interest

The authors declare that the research was conducted in the absence of any commercial or financial relationships that could be construed as a potential conflict of interest.
